# Chinese Singaporean Children's Expectations About Peer Group Norms in the Context of Wealth and Ethnicity

**DOI:** 10.1111/desc.70175

**Published:** 2026-03-24

**Authors:** Alexandra Paquette, Leher Singh, Marley B. Forbes, Melanie Killen

**Affiliations:** ^1^ Department of Psychology National University of Singapore Singapore; ^2^ Department of Psychology University of Maryland Baltimore County Baltimore Maryland USA; ^3^ Department of Human Development and Quantitative Methodology, University of Maryland College Park College Park Maryland USA

**Keywords:** ethnic bias, peer inclusion, prejudice, wealth status

## Abstract

United Nations SDG 10 focuses on reducing inequalities in civil society. To address the emergence of attitudes and reasoning about social exclusion based on wealth and ethnicity, Chinese Singaporean children's predictions about peer group inclusion decisions based on ethnicity (Chinese/Indian) and wealth (high/low) were investigated. A cohort of 103 participants (4–7 years, *M*_age_ = 5.79; 49% female) was asked to predict and reason about how a peer group would make choices about including a new peer over three conditions. In the Ethnicity condition, participants expected peers to give priority to same‐ethnicity friendships; this expectation increased with age. In the Wealth condition, participants’ expectations for peers to give priority to same‐wealth friendships increased with age. When both ethnicity and wealth were independently manipulated, participants expected their peers to give priority to high wealth, even if that meant the group would be cross‐ethnic. Children referenced access to material resources and opportunities when reasoning about why low‐wealth and high‐wealth groups would choose to include a high‐wealth peer. In same‐wealth contexts, shared identity between the peer and group was also referenced. Participants used moral reasoning to explain their expectations for the low‐wealth Chinese group's choice of a high‐wealth Chinese peer; this reasoning was not evidenced when Chinese participants predicted a low‐wealth Indian group's choice for including a high‐wealth Indian peer. These novel findings revealed children's nuanced expectations about exclusionary peer group norms regarding wealth and ethnicity. Reducing exclusionary attitudes early in life contributes to sustainable development across cultural contexts.

**Summary:**

- Young Chinese Singaporean children expected peer groups to include high‐wealth peers, though older children increasingly predicted low‐wealth groups would include peers of the same wealth.
- Children expected groups to prioritize high wealth over shared ethnicity when both traits were presented simultaneously.
- Children reasoned that groups include high‐wealth peers due to access to material resources and, at times, shared group identity.
- Moral reasoning emerged when children predicted that low‐wealth Chinese, but not Indian, groups would include high‐wealth peers for resource‐sharing purposes.

## 1 Introduction

United Nations SDG 10 focuses on reducing inequalities in civil society. To achieve this goal, developmental scientists need to understand the origins of bias and social exclusion, which are common sources of social inequality. Extensive research has revealed that various forms of bias emerge in childhood and begin to negatively affect targets of such exclusion. For example, experiencing exclusion in childhood can negatively impact one's physical and mental health, and overall well‐being (Cheng et al. 2015; Currie et al. 2019; Forrest‐Bank and Jenson 2015). As both targets, perpetrators, and witnesses to bias and social exclusion, children hold nuanced perspectives on the acceptability of such behaviors. Prior research on children's views of social exclusion has largely focused on children's evaluations of whether it is acceptable to include or exclude others based on single group categories such as gender, race, ethnicity, and religion (Rutland et al. 2024). This study extends efforts to understand the developmental origins of bias by investigating two intersecting forms of exclusion that jointly contribute to social inequality—ethnicity‐based and wealth‐based exclusion.

An increasing number of recent studies have considered how children evaluate exclusion based on wealth and social status, illustrating that children also demonstrate early preferences based on these factors (Ahl et al. 2019; Burkholder et al. 2021; Elenbaas 2019; Horwitz et al. 2014; Shutts et al. 2016). However, the intersection of ethnicity and wealth bias has received little attention, despite these variables being interrelated in most societal contexts. Researchers have asserted that studies should assess biases and perceptions based on multiple group identities given the intersectional nature of many social categories (Lei and Rhodes 2021). Additionally, most previous research on social exclusion based on wealth has been conducted in US contexts, leaving the generalizability of these findings to other global contexts unknown. As wealth inequalities and social exclusion based on one's socioeconomic status occur globally, more research is warranted to consider how conceptions of wealth and ethnicity emerge in childhood in a range of countries and contexts.

Although children have been shown to hold individual preferences for inclusion based on wealth and ethnicity, less is known about young children's expectations of group norms about inclusion decisions. Investigating children's expectations about group norms pertaining to inclusion is distinct from examining children's evaluations of acts of inclusion (i.e., whether the act is legitimate or not). Expectations about what a group would do provides a window into children's knowledge of group norms and group‐generated biases, whereas evaluations provide insight into children's judgments of acceptability or fairness. Thus, the present study investigated the emergence of expectations about group norms pertaining to inclusion of peers based on ethnicity and wealth in young Chinese children growing up in Singapore, an island country and city‐state in Southeast Asia with a numeric majority Chinese and minority Indian ethnic composition.

The distinction between race and ethnicity is often ambiguous, and usage varies across contexts and over time. In Singapore, race and ethnicity are frequently used interchangeably. The same state‐defined groupings (Chinese, Malay, Indian, and Other) are officially classified as racial categories, while comparable groupings are treated as ethnic divisions in Malaysia (Hirschman 1987; Rocha and Yeoh 2022). This is further reflected in Singapore's “Ethnic Integration Policy,” which, despite its name, is described by the government as promoting “racial” integration (HDB's Ethnic Integration Policy 2020). Together, these examples illustrate the fluidity of the race‐ethnicity boundary across sociocultural settings. In the present paper, we use the term ethnicity for consistency, while acknowledging that both terms are historically and contextually defined. We use the term race only when referring to prior research that employed that terminology.

### 1.1 Perceptions of Ethnicity and Wealth in Childhood

Studies with older children have examined evaluations of exclusion based on ethnicity, finding that children and adolescents view ethnicity‐based peer exclusion negatively, with this tendency increasing with age (Killen et al. 2010; Ruck et al. 2010; Thijs 2017). Research in the United States further indicates developmental and group differences in awareness of ethnic bias, with African American and Latino children often showing earlier sensitivity to ethnic bias than White children (Brown et al. 2011). More recent work with children aged 6–12 years found that children who rejected same‐race inclusion justified by parental preference used moral reasoning focused on fairness and equal treatment to explain their evaluation (Luken Raz et al. 2025).

When forming expectations about social inclusion and exclusion, older children have also been shown to consider wealth alongside ethnicity. With increasing age, US 8‐ to 14‐year‐olds expected high‐wealth groups to be more exclusive than low‐wealth groups, regardless of depicted race (Burkholder et al. 2020). When interviewing participants about inclusion, Burkholder et al. (2021) found that, with age, White US children expected that clubs would include a same‐wealth peer (even when this peer was a different race). In contrast, with age, Black US children expected that Black peers would include a same‐ethnic peer (even when this peer was of a different wealth level). A study with children ages 8–10 and 14–16 years living in Türkiye revealed that adolescents, more than children, viewed their peers’ exclusion based on wealth status as wrong due to unfair treatment (Gönül et al. 2023). Collectively, these studies demonstrate that, with age, children make decisions about others based on contextual considerations of race, ethnicity, and wealth.

Although research with older children has examined evaluations and expectations of ethnic‐ and wealth‐based exclusion, much less is known about how younger children reason about peer inclusion and exclusion in everyday contexts. Existing work with preschool‐aged children has instead largely focused on social preferences and biases about one attribute, such as ingroup preferences based on race and ethnicity (Gonzalez et al. 2017; Perszyk et al. 2019; Qian et al. 2015, 2019; Xiao et al. 2014). Separately, biases based on wealth and social class have also been documented in preschool‐aged children. Shutts et al. (2016) investigated social class bias in preschool children, demonstrating that children preferred high‐wealth children over low‐wealth children. Moreover, children form early stereotypes about wealth, such as associating high wealth with generosity and benevolence (Ahl et al. 2019). Children's perceptions of themselves as high or low wealth can also moderate their wealth bias, as Horwitz et al. (2014) demonstrated that children who were assigned to membership in high‐wealth groups showed more ingroup favoritism than those assigned to low‐wealth groups.

Importantly, children's conceptions of ethnicity and wealth do not exist fully independently. For example, Indonesian children reliably associated Chinese Indonesians with wealth and Native Indonesians with political influence and displayed a preference for Chinese Indonesians by 6.5 years of age (Amemiya et al. 2023). Thus, while it is clear that children begin to use ethnicity and race as cues for social status early in childhood, less work has studied young children's expectations of others’ group norms about wealth and ethnicity, despite recent findings with older children.

### 1.2 The Role of Group Norms in Peer Inclusion and Exclusion

Starting in the preschool years, children exhibit an emerging understanding of group dynamics which encompass recognizing group norms (shared beliefs and traditions) and differentiating them from group membership (ingroup and outgroup affiliations) (Killen et al. 2018; Nesdale and Lawson 2011). Group dynamics influence how children evaluate peer inclusion and exclusion, with decisions often shaped by their identification with social categories, such as ethnicity and wealth, as well as group norms regarding acceptable behaviors (Burkholder et al. 2021; Olson et al. 2012). Age‐related changes are evident, as younger children (3–4 years old) tend to rely on ingroup biases and stereotypes, while older children (5–6 years old) begin to critically evaluate group norms, particularly when they conflict with concerns about fairness and equality (Killen et al. 2018).

Yet, even when children individually disagree with group norms that conflict with moral principles, challenging group norms that perpetuate bias and unfair treatment within peer groups is a difficult decision for children to make. Such decisions are difficult for children largely due to their concerns about how groups will react if their norms are challenged. By middle childhood, children begin to understand that peer groups may perceive challenges to their norms as threats to their group functioning and identity, and these threats may in turn cause the group to retaliate against the challenger and exclude them from the peer group (Mulvey et al. 2014; Rutland et al. 2015). These concerns about the costs of challenging group norms strengthen as children enter adolescence and can serve as a barrier to standing up against bias and unfair treatment (Barth et al. 2025; Yüksel et al. 2025). Ultimately, this understanding of group norms plays a critical role in shaping how children balance concerns for fairness and equality with group expectations and preferences when evaluating or predicting inclusion and exclusion decisions in intergroup contexts.

### 1.3 Social Context of Singapore

Singapore is a multi‐ethnic country with three major ethnic groups: Chinese (75.9%), Malay (15.0%), and Indian (7.5%) (Department of Statistics Singapore 2020). Moreover, the government has put in place policies to encourage ethnic harmony and integration (Lee and Setoh 2022). For instance, the Housing and Development Board (HDB), which is responsible for public housing that approximately 80% of the population resides in (Department of Statistics Singapore 2023), has an Ethnic Integration Policy that mandates that in each block or neighborhood, there must be a minimum representation of residents from each of the three major ethnic groups (HDB's Ethnic Integration Policy 2020).

Nonetheless, studies conducted in Singapore have illustrated that Chinese children ages 3–7 exhibit implicit and explicit ethnic biases favoring their ingroup (Setoh et al. 2019). Lee and Setoh (2022) demonstrated that the ability to categorize novel faces by ethnicity in early childhood increases with age. Specifically, they found that 3‐ to 4‐year‐olds did not exhibit any own‐ethnicity preferences, whereas 5‐ to 6‐year‐olds did. The expression of bias was related to children's abilities to categorize other people's faces: those with better abilities to categorize individuals based on ethnicity showed more bias.

These studies point to the presence of ethnicity bias in preschool children in Singapore, also found in other countries (e.g., Gaias et al. 2018; Qian et al. 2015). However, an interesting and novel pattern has emerged in Singapore based on participants’ ethnicity. Setoh et al. (2019) demonstrated that while Chinese Singaporean preschool children demonstrated an ingroup bias, Indian Singaporean children had no implicit or explicit ingroup bias, even though both groups were able to categorize children based on their ethnic background. This suggests that the expression of bias in Singapore depends on children's ethnic background and that ingroup biases vary by one's own position in society. Thus, we aimed to investigate whether children expected their ethnic group and other ethnic groups to display an in‐group preference in the context of wealth status, and how they reason about these decisions.

### 1.4 Relationship Between Ethnicity and Wealth Biases in Singapore

Singapore is a high‐wealth economy and is ranked one of the eighth richest countries in the world (World Bank 2019). In Singapore, Chinese and Indians receive approximately the same average salaries, with Malays earning less. Although Indians and Chinese reflect a similar level of wealth (Vergis 2023), negative attitudes exist toward Indians in Singapore in the form of discrimination and bias (Velayutham 2016; Woods and Kong 2023). Our stimulus selection was motivated by the existence of bias toward Indians in Singapore despite the fact that wealth inequalities are not significantly different and that prior studies conducted in Singapore have demonstrated an ingroup preference for Chinese adults over Indian adults in social learning paradigms (Singh et al. 2019; see also Singh et al. 2020). As well, studies with children have demonstrated that from an early age, Singaporean Chinese children visually distinguish between individuals of Chinese and Indian descent (Singh et al. 2022). Further, as preschoolers, Singaporean Chinese children demonstrate both implicit and explicit bias against Indian adults (Setoh et al. 2019; see also Singh, Quinn et al. 2020). Therefore, there is prior evidence that Singaporean Chinese children express bias toward adults of Indian origin, but no research has demonstrated whether this bias generalizes to images of Indian children (not adults) and manifests as expectations that peers will show in‐group bias though peer inclusion decisions.

An understudied topic is how Singaporean children conceptualize wealth status and ethnic background in the context of peer groups. Although other social categories such as religion and gender are also salient in Singapore, we focused on ethnicity and wealth because these two dimensions are strongly intertwined and likely shape children's social expectations. The few studies conducted in Singapore have focused on racial categorization as measured by preferences for faces that vary by race or ethnicity, which is an important demonstration of biases among young children in this context (Lee and Setoh 2022). No studies that we know of have examined how Singaporean children make predictions about inclusion for peer groups when wealth status or ethnic background is considered. Expectations about group preferences regarding social inclusion of peers from different backgrounds provides an important window into children's perceptions of what counts as normative behavior (Cooley and Killen 2015). Thus, children's predictions about peer group inclusion based on wealth status and ethnic background were the focus of our study.

### 1.5 Theoretical Framework

The theoretical framework guiding this study was the social reasoning developmental (SRD) model (Elenbaas et al. 2020), which draws upon both social identity theory (Nesdale 2008) and social domain theory (Smetana and Yoo 2023) to understand children's social cognition about issues of peer inclusion and exclusion in intergroup contexts. Developmental scientists have demonstrated that in‐group bias and out‐group distrust exist in childhood, and particularly when social comparisons and categorization processes emerge (Ahl et al. 2019; Horwitz et al. 2014). The SRD model posits that children coordinate moral (fairness), group‐based (shared group identity), and psychological (personal choice) concerns when making decisions and predictions in contexts where social group membership is salient. Specifically, reasoning based on fairness, equity, and others’ welfare is often used when making inclusive decisions, while reasoning based on shared group identity and group norms is often used to justify prejudicial attitudes and behaviors (Rutland et al. 2024).

Most of the research on children's expectations of peer group preferences has focused on older children, typically those in middle childhood or early adolescence (e.g., Burkholder et al. 2020, 2021; Grütter et al. 2021; Gönül et al. 2023). In contrast, our study examines younger children, ages 4–7, a developmental period that has been less explored in the context of group norms and expectations of group inclusion decisions. This is also a period when age‐related changes are emerging: younger children tend to rely more on ingroup biases, whereas slightly older children begin to consider fairness and group norms (Killen et al. 2018). Currently, what we know about bias with young children is whether they display a negative bias about someone from a different racial and ethnic background (Qian et al. 2015). Yet, displaying a bias and making predictions about what other children in a group would do is different. Examining young children's expectations for how groups include peers based on ethnicity or wealth provides insights into their early understanding of social status, hierarchies, and group dynamics. Investigating these early beliefs about social status preferences has the potential to yield important information for how to design programs to reduce biases and facilitate intergroup friendships.

### 1.6 The Present Study

In the present study, we investigated how Chinese Singaporean children made predictions and reasoned about peer group social inclusion decisions regarding ethnicity (Chinese, Indian) and wealth status (high, low). There were three conditions: Ethnicity, Wealth, and Cross Ethnicity/Wealth. In the Ethnicity condition, children were presented with photos displaying four same‐ethnic (Chinese) peers and four other‐ethnic (Indian) peers. For each group, they were presented with photos of one Chinese and one Indian child, and then asked which child they thought the group would choose and why. In the Wealth condition, children were shown a high‐wealth Chinese group and a low‐wealth Indian group. For each group, they were presented with a high‐wealth and low‐wealth child that matched the group's ethnicity and asked which child they thought the group would choose and why. In the Cross Ethnicity/Wealth condition, children were presented with a high‐wealth Chinese or a low‐wealth Chinese group. For each group, they were presented with a *same‐ethnic other‐wealth* child and a *same‐wealth other‐ethnic* child. Participants were asked which child they thought the group would choose to include and why.

### 1.7 Hypotheses

Based on the SRD model (Elenbaas et al. 2020) and previous research (Burkholder et al. 2021), we hypothesized that participants would make different predictions about peer inclusion for ethnicity and wealth. Regarding ethnicity, we predicted that, for both groups, participants would predict that the group would choose the same‐ethnic peer (H1a). This is because when given no other information, children will focus on the same ethnicity or race as a factor to make predictions on peer group decisions about inclusion (McGlothlin et al. 2005; Roberts et al. 2017). In particular, children from high‐status groups—such as the majority‐ethnic Chinese participants in our study—are more likely to view same‐ethnicity inclusion as the norm compared to interracial inclusion (Cooley et al. 2019). We also predicted that this expectation of a preference for a same‐ethnicity peer would increase with age (H1b), as children have been shown to display a stronger preference for same‐ethnicity affiliation over time (Cooley et al. 2019; Hitti and Killen 2015).

Next, we hypothesized that participants would predict that both a high‐ and low‐wealth group would choose the high‐wealth peer for inclusion when the ethnicity of peers was held constant given previous research on young children's own high‐wealth preferences (Shutts et al. 2016) and the emphasis on high wealth in Singapore (H2a). However, we also predicted age related differences: with age, participants would increasingly expect groups to prioritize selecting a same‐wealth peer (H2b). This expectation was grounded in prior research with older children and adolescents demonstrating that they often give priority to shared wealth status when making peer inclusion decisions (Burkholder et al. 2021).

When varying wealth and ethnicity, we predicted that participants would expect a low‐wealth Chinese group to choose a high‐wealth Chinese peer to be in their group (same‐ethnic but *other‐wealth*) while also expecting a high‐wealth Chinese group to select a high‐wealth Indian peer (same‐wealth but *other‐ethnic*) to be in their group (H3). These expectations were based on theories that wealth is viewed as a high‐status variable that children become aware of very early (Heck et al. 2022), and particularly in a country like Singapore which emphasizes its high wealth status (World Bank 2019). This hypothesis was distinct from previous research on wealth and ethnicity which has shown that children expect low‐wealth groups to include same‐wealth peers (e.g., Burkholder et al. 2021); here it was predicted that children would expect low‐wealth groups to include high‐wealth peers. We did not make a prediction for age‐related effects in the condition where both wealth and ethnicity were varied, as there is insufficient prior research to indicate how children's expectations might change with age.

Finally, we made several predictions with regards to children's reasoning for their expectations. First, we expected that participants would frequently reference extra material resources and shared identity when predicting why high‐wealth groups would choose high‐wealth members, given the salience of materialism and desire for wealth in Singapore (Peng 2019) and given that Burkholder et al. (2021) found that participants most often cited shared similarity and comfort when explaining why a group would pick a same‐wealth peer (H4a). Next, we predicted that young children would most frequently reference extra material resources and moral reasons when predicting why low‐wealth groups would choose high‐wealth members (H4b). This was based on previous research demonstrating that young children anticipate resource‐rich individuals will share with others (Ahl and Dunham 2019).

## 2 Methods

### 2.1 Participants

Participants included 103 4‐ to 7‐year‐olds (*M* = 5.79, *SD* = 1.07, 49% female) recruited in Singapore from a university laboratory database where parents interested in participating in research studies with their children signed up to volunteer. The sample was drawn from an urban environment. All participants were of Chinese ethnicity. Participants were mostly from middle‐ to upper‐middle‐class families as reported by their parents using the MacArthur Scale of Subjective Social Status (*M* = 6.13, *SD* = 1.10, range: 3–10). Most participants’ mothers (83%) and fathers (76%) held a bachelor's degree or higher. The majority of participants (92%) reported having same‐ethnic Chinese friends (see Supplemental Materials for details).

### 2.2 Procedure

The study was approved by the Departmental Ethics Review Committee (DERC) at the National University of Singapore. Informed consent was obtained from the children's parents, and verbal assent was obtained from the children. Participants were individually interviewed in a quiet and private room in a university lab or at their home by the first author, who was born and raised in Singapore. One additional member of the research team, a long‐term Singapore resident, was based in Singapore during the study's design phase, and overall, half of the author team were long‐term residents or citizens of Singapore, supporting the cultural expertise of the research team.

Participants were seated next to the experimenter and throughout the experiment, they were shown photos of children and visual representations of wealth on a laptop screen via a PowerPoint presentation. The interview lasted approximately 30–40 min. The data were collected in 2022.

There were three conditions in the experiment: Ethnicity, Wealth, and Cross Ethnicity/Wealth conditions (see Table 1). The three conditions were always presented in a fixed order (Ethnicity, Wealth, Cross). By presenting the Ethnicity condition first, we aimed to isolate the effects of ethnicity without the potential confounding influence of wealth considerations. All participants viewed both groups in the Ethnicity (Chinese, Indian) and Wealth (high‐wealth, low‐wealth) conditions. Within the Ethnicity and Wealth conditions, the order of the scenarios was counterbalanced across participants (e.g., for the Ethnicity condition, half of participants saw a Chinese group first, while the other half saw an Indian group first). For the Cross Ethnicity/Wealth condition, participants were randomly assigned to view and respond to items about only one condition (low‐wealth Chinese or high‐wealth Chinese). In each condition, participants were initially introduced to a school and to a group of children who played together after school. Participants were told that the group could choose one more child to join them to play. The two friends differed in ethnicity (Ethnicity condition), wealth (Wealth condition), or both ethnicity and wealth (Cross Ethnicity/Wealth condition).

**TABLE 1:** Descriptions of the friend group and the two characters in each scenario in each condition.

Condition	Friend group	Characters to Include
Ethnicity	Chinese	1. Chinese 2. Indian
	Indian	1. Chinese 2. Indian
Wealth	High‐wealth Chinese	1. High‐wealth Chinese 2. Low‐wealth Chinese
	Low‐wealth Indian	1. High‐wealth Indian 2. Low‐wealth Indian
Cross	High‐wealth Chinese	1. Low‐wealth Chinese 2. High‐wealth Indian
	Low‐wealth Chinese	1. High‐wealth Chinese 2. Low‐wealth Indian

*Note*: Participants were presented conditions in a fixed order (Ethnicity, Wealth, then Cross). For the Ethnicity and Wealth conditions, participants responded to both friendship groups (Chinese and Indian for Ethnicity; High‐wealth Chinese and Low‐wealth Indian for Wealth). For the Cross condition, participants were randomly assigned to respond to either the High‐wealth Chinese or Low‐wealth Chinese friendship group.

The general methodological approach was inspired by the approach taken by Burkholder et al. (2021) and adapted for younger children in a different developmental and cultural context to examine the early emergence of reasoning about ethnicity and wealth in expectations for friendship group inclusion decisions.

### 2.3 Measures

The peer groups were represented by photographs of four individual children (two girls and two boys). The two new friends were similarly represented by photographs of individual children and were matched to the participant's gender. All photographs were rated by 10 Singaporean Chinese adults and confirmed to be representative of their ethnic group, as well as matched for friendliness and attractiveness (see Supplemental Materials).

In the Wealth and Cross Ethnicity/Wealth conditions, we followed previous studies on wealth bias (Burkholder et al. 2020; Elenbaas 2019; Gönül et al. 2023; Olson et al. 2012) where high‐wealth and low‐wealth groups and new friends were presented with images of wealth within their cultural context which often included a poor or rich house, new or old car, and other images. For the Singapore context for high wealth, we included photos of a stack of many dollar bills, a large, landed property home, a nice car, and a beach vacation. The landed property home depicted is a significant symbol of affluence in Singapore, where less than 5% of the population resides in such exclusive housing (Department of Statistics Singapore 2023). Additionally, car ownership is a prominent symbol of affluence as only about one third of the Singaporean population owns cars due to their high expense (Loi 2023). The high‐wealth groups and friends were introduced as follows: “Their family has a lot of money. They live in a house like this, ride in a car like this, and during the holidays, they go on vacations to the beach.”

**FIGURE 1:**
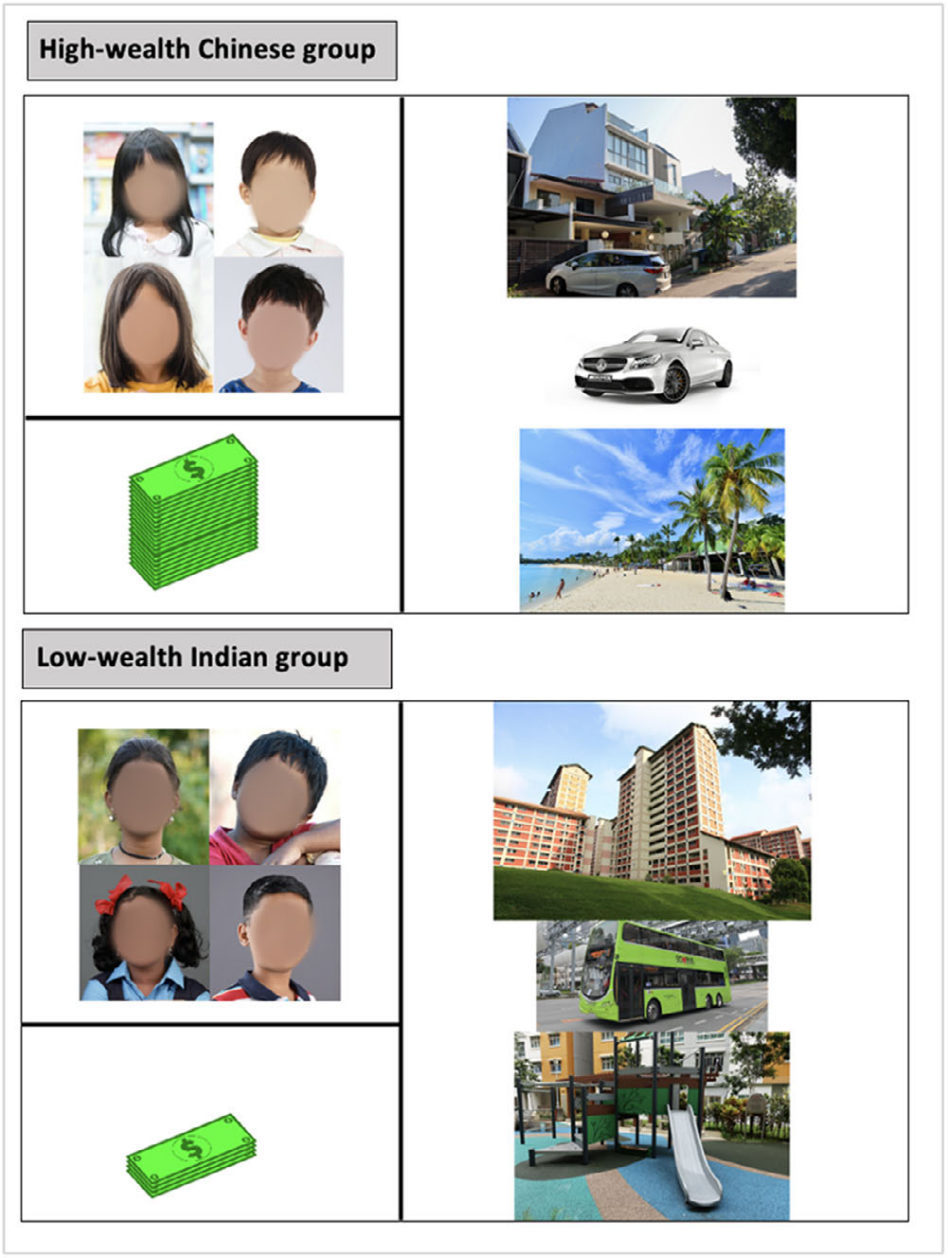
Example of visual stimuli shown when introducing high‐ and low‐wealth groups. Faced are blurred here for publication purposes only; the original stimuli shown to participants contained unblurred images. The pictures represent the house, type of transportation, and type of vacation associated with each income level. High‐wealth groups and peers were depicted with a landed property home, expensive car, and a beach vacation. In contrast, low‐wealth groups and peers were depicted with a modest public housing building, a public bus, and a simple neighborhood playground.

For the low‐wealth context, we included photos of a stack of a few dollar bills, a modest public housing apartment building, a public bus, and a simple public playground. Public housing in Singapore, known as HDB (Housing Development Board) flats, accommodates approximately 80% of the population (Department of Statistics Singapore 2023) and is the most affordable form of housing. Riding the public bus is a common mode of transportation in Singapore, particularly for lower income households who may not own cars or who live farther from metro stations, where housing is more expensive (Begum 2022). The low‐wealth groups and friends were introduced as follows: “Their family has a little bit of money. They live in a house like this, ride the bus, and during the holidays, they stay at home and play outside.” (See Figure 1.)

**Choice Predictions**. After being introduced to each group of friends and the two new characters, participants were asked, “Who do you think this friend group would choose to join them?” For the Ethnicity condition, participants responses were recorded as prediction of other‐ethnic (0) or same‐ethnic (1). For the Wealth condition, participants responses were recorded as prediction of other‐wealth (0) or same‐wealth (1). For the Cross Ethnicity/Wealth condition, participants responses were recorded as prediction of same‐wealth other‐ethnic (0) or same‐ethnic other‐wealth (1).

**Reasoning**. Children's reasoning for their choice predictions was assessed by asking, “Why?” after children gave their predictions. Reasoning categories were derived from previous research on evaluations of wealth status and pilot testing (e.g., Burkholder et al. 2021). Coding categories included: (1) moral reasoning; (2) shared ethnicity and skin color; (3) shared identity regarding wealth; (4) shared identity regarding language or traditions; (5) positive physical appearance; and (6) recognition of extra material resources (see Table 2 for more information about the coding categories). Responses that did not fit any of the six categories (e.g., “I don't know”) were coded as Uncodable. Following previous research (Elenbaas 2019), reasoning was coded as proportions by the experimenter and by a second coder who was blind to its hypotheses. Full use (coded as 1) indicates that participants solely relied on the given category to justify their preferences. Partial use (coded as 0.5) indicates that participants incorporated elements from two categories into their reasoning. No use (coded as 0) indicates that participants did not incorporate the provided categories into their reasoning at all. There were no cases in which responses contained elements from three or more categories. Based on approximately 20% of the interviews (*n *= 20), Cohen's κ = 0.86 for interrater reliability for all categories was achieved. Disagreements between coders were resolved through discussion until consensus was reached.

**TABLE 2:** Reasoning coding system.

Conceptual category	Definition	Example
Moral reasoning (fairness, sharing, benefits of diversity)	References to the unfairness of different levels of wealth, the positive outcomes of sharing and/or the benefits of having a diverse group	“Because she has more money, so she can share the money with the group.”
Shared ethnicity and/or skin color	References to characters sharing the same skin color	“Because he's the same skin color.”
Shared identity regarding wealth	References to characters sharing the same level of material resources	“Because she got less money and they got less money too.”
Shared traditions, customs, and language	References to characters sharing traditions, customs, interests, and/or language	“I think they have the same language.”
Positive physical appearance	References to characters’ physical appearance not related to skin color	“He has a nice smile.”
Recognition of excess or lack of material wealth and opportunities	References to characters’ excess or lack of material resources and/or opportunities	“They have a lot of money so they can buy a lot of things and go for holiday.”

### 2.4 Data Analytic Strategy

#### 2.4.1 Choice Predictions

Children's predictions for which peer they expected the group to choose to join them were dichotomous: same‐ or other‐ethnic friend for the *Ethnicity* condition, same‐ or other‐wealth friend for the *Wealth* condition, and same‐ethnic other‐wealth or same‐wealth other‐ethnic for the *Cross* condition. As a result, the predictions were analyzed with logistic regression models.

For the Ethnicity and Wealth conditions, we analyzed participants’ responses with linear mixed‐effects logistic models. As predictors, we included children's age in years and the group's identity (Chinese or Indian for *Ethnicity* condition, high‐ or low‐wealth for the *Wealth* condition). All predictors were mean‐centered. Given the repeated‐measures design (i.e., each participant responded to the same item for two groups in each condition), we included a random intercept for participant in our models (Muradoglu et al. 2023).^1^

For the Cross condition, participants’ responses were analyzed with a binomial logistic regression model, as there was no repeated‐measures design (i.e., participants only saw either the high‐wealth Chinese or low‐wealth Chinese group in this condition). As predictors, we included children's age in years and condition (high‐wealth Chinese or low‐wealth Chinese).

All analyses were conducted in R statistical software Version 4.4.2 (R Core Team 2024). For the mixed‐effects models, the packages *lme4* (Version 1.1‐35.5, Bates et al. 2015) and *lmerTest* (Version 3.1.3, Kuznetsova et al. 2017) were used. For all logistic models, odd ratios (*OR*) are reported as effect size measures. A simulation‐based power analysis was conducted with the *simr* package (Green and MacLeod 2016), which confirmed that a sample size of at least 100 would be sufficient to detect the expected fixed effects at 80% power and alpha = 0.05.

#### 2.4.2 Reasoning

Children's reasoning responses explaining why they made their predictions were analyzed using one‐way repeated measures ANOVAs. Follow‐up *t*‐tests with Bonferroni corrections were conducted to clarify significant results. For each condition, analyses were run on the three most frequently used reasoning categories, in line with similar past research (Mulvey et al. 2014; Gönül et al. 2023). Reasoning for the *Ethnicity* condition was not analyzed because more than half (53%) of responses were uncodable. Thus, only reasoning data from the *Wealth* and *Cross* conditions were analyzed.

## 3 Results

### 3.1 Choice Predictions

#### 3.1.1 Ethnicity Condition

As hypothesized (H1a), children's predictions that groups would choose a same‐ethnic peer to join them (*M* = 0.66, *SD* = 0.47) were significantly greater than chance, as indicated by the statistically significant intercept in the model, *b* = 1.13 [0.46, 1.81], *SE* = 0.35, *p* = 0.001, *OR* = 3.11. The odds of predicting that the group would choose a same‐ethnic peer were 3.11 times the odds of predicting that the group would choose an other‐ethnic peer.

Age had a significant effect on children's predictions that groups would choose a same‐ethnic peer to join them, *b* = 0.70 [0.13, 1.26], *SE* = 0.29, *p* = 0.015, *OR* = 2.01. Specifically, the odds of predicting that the group would choose a same‐ethnic peer increased by a factor of 2.01 for every year increase in age. This finding suggests that, with age, children are more likely to expect peer groups to hold preferences for same‐ethnicity peers, confirming H1b.

The effect of the group's ethnicity on children's predictions that groups would choose a same‐ethnic peer to join them was not significant, *b* = −0.50 [−1.26, 0.026], *SE* = 0.39, *p* = 0.195, *OR *= 0.61. The extent to which children predicted that a Chinese group (*M *= 0.69, *SD* = 0.47) and an Indian group (*M *= 0.62, *SD* = 0.49) would choose a same‐ethnic peer did not significantly differ (see Table 3).

**TABLE 3:** Mixed‐effects logistic model predicting children's expectation of a group choosing a same‐ethnic peer in the Ethnicity condition.

Fixed effects	*b*	*SE*	*p*	95% CI	
Intercept	**1.13**	**0.35**	**0.001**	**0.46**	**1.81**
Group's ethnicity	−0.50	0.39	0.195	−1.26	0.26
Age	**0.70**	**0.29**	**0.015**	**0.13**	**1.26**

Random effects	*Variance*				
Participant intercept	3.43				

*Note*: Bolded coefficients are statistically significant (*p* < 0.05).

#### 3.1.2 Wealth Condition

Overall, children's predictions that groups would choose a same‐wealth peer to join them (*M* = 0.60, *SD* = 0.49) were significantly greater than chance, as indicated by the significant intercept in the model, *b* = 0.52 [0.16, 0.89], *SE* = 0.19, *p* = 0.005, *OR* = 1.69. The odds of predicting that the group would choose a same‐wealth peer were 1.69 times the odds of predicting that the group would choose an other‐wealth peer.

Although children's predictions overall reflected an expectation that groups would choose a same‐wealth peer, predictions for the high‐wealth Chinese group to choose a same‐wealth peer (*M* = 0.80, *SD* = 0.40) differed from predictions for the low‐wealth Indian group to choose a same‐wealth peer (*M* = 0.40, *SD* = 0.49). The effect of the group's wealth on children's predictions that the group would choose a same‐wealth peer was significant, *b* = −1.98 [−2.78, −1.18], *SE* = 0.41, *p *< 0.001, *OR* = 0.14. Specifically, the odds of predicting that the low‐wealth Indian group would choose a same‐wealth peer were 0.14 times the odds of predicting that the high‐wealth Chinese group would choose a same‐wealth peer. This result indicates that children expected a high‐wealth group's preference for a same‐wealth peer to be greater than a low‐wealth group's preference for a same‐wealth peer, thus partially supporting H2a.

As hypothesized (H2b), age also had a significant effect on children's predictions that groups would choose a same‐wealth peer to join them*, b* = 0.36 [0.03, 0.70], *SE* = 0.17, *p* = 0.032, *OR* = 1.44. Specifically, the odds of predicting that the group would choose a same‐wealth peer increased by a factor of 1.44 for every year increase in age (see Table 4).

**TABLE 4:** Mixed‐effects logistic model predicting children's expectation of a group choosing a same‐wealth peer in the Wealth condition.

Fixed effects	*b*	*SE*	*p*	95% CI	
Intercept	**0.52**	**0.19**	**0.005**	**0.16**	**0.89**
Group's wealth	**−1.98**	**0.41**	**<0.001**	**−2.78**	**−1.18**
Age	**0.36**	**0.17**	**0.032**	**0.03**	**0.70**

Random effects	*Variance*				
Participant intercept	0.38				

*Note*: Bolded coefficients are statistically significant (*p* < 0.05).

#### 3.1.3 Cross Condition

Overall, children predicted that the group would choose the same‐ethnic other‐wealth peer approximately half of the time (*M* = 0.51, *SD* = 0.50). However, predictions for the group's choice of the same‐ethnic other‐wealth peer varied by condition, with a majority of participants in the low‐wealth Chinese group condition (*M* = 0.76, *SD* = 0.43) predicting that the group would choose the same‐ethnic other‐wealth peer, and a minority of participants in the high‐wealth Chinese group condition predicting that the group would choose the same‐ethnic other‐wealth peer (*M* = 0.27, *SD* = 0.44).

The effect of condition on predictions that the group would choose the same‐ethnic other‐wealth peer was significant, *b* = 2.21 [1.30, 3.12], *SE* = 0.46, *p* < 0.001, *χ* ^2^(1) = 22.7, *OR* = 9.10. Specifically, the odds of expecting the group to choose the same‐ethnic other‐wealth peer were 9.10 times higher in the low‐wealth Chinese group condition compared to the high‐wealth Chinese group condition. This finding suggests that children expected both peer groups to have a high‐wealth preference, even if that meant choosing an other‐ethnic peer, such as in the case of the high‐wealth Chinese group, therefore supporting H3.

The effect of age was not significant, *b* = −0.32 [−0.76, 0.12], *SE* = 0.28, *p* = 0.256, *OR* = 0.73, indicating that children's likelihood of expecting the group to pick a same‐ethnic other‐wealth peer did not vary significantly with age (see Table 5 for details on the model; see Table 6 for a summary of children's predictions about groups’ choice of peers across all three conditions).

**TABLE 5:** Binomial logistic regression model predicting children's expectation of a group choosing a same‐ethnic other‐wealth peer in the Cross condition.

Fixed effects	*b*	*SE*	*p*	95% CI	
Intercept	0.84	1.30	0.516	−1.70	3.39
Condition	**2.21**	**0.46**	**<0.001**	**1.30**	**3.12**
Age	−0.32	0.22	0.149	−0.76	0.12

*Note*: Bolded coefficients are statistically significant (*p* < 0.05).

**TABLE 6:** Summary of children's predictions about which peer they expected groups to choose to invite to join them.

Condition	Friendship groups’ characteristics	New friends’ characteristics	Participants’ overall expectations for the group's choice of the new peer
Ethnicity	Chinese	1. Chinese 2. Indian	Same‐ethnic (Chinese) friend
	Indian	1. Chinese 2. Indian	Same‐ethnic (Indian) friend
Wealth	High‐wealth Chinese	1. High‐wealth Chinese 2. Low‐wealth Chinese	Same‐wealth (high‐wealth Chinese) friend
	Low‐wealth Indian	1. High‐wealth Indian 2. Low‐wealth Indian	Other‐wealth (high‐wealth Indian) friend
Cross (Ethnicity/Wealth)	High‐wealth Chinese	1. High‐wealth Indian 2. Low‐wealth Chinese	Same‐wealth but *other‐ethnic* (high‐wealth Indian) friend
	Low‐wealth Chinese	1. High‐wealth Chinese 2. Low‐wealth Indian	Same‐ethnic but *other‐wealth* (high‐wealth Chinese) friend

*Note*: For choice measures, participants were asked, “Who do you think this friend group will choose to join them?”.

### 3.2 Reasoning

#### 3.2.1 Wealth Condition

Participants first viewed a high‐wealth Chinese group who could choose a same‐wealth (high‐wealth) Chinese friend, or an other‐wealth (low‐wealth) Chinese friend. Overall, participants expected the group to prefer a same‐wealth (high‐wealth) friend. A one‐way repeated measures ANOVA was performed to compare the usage of the three most frequently used reasoning categories (moral reasoning, shared identity regarding wealth, recognition of extra material resources) by participants who demonstrated this expectation. The repeated measures ANOVA revealed a significant difference between the reasoning categories used, *F*(2) = 15.66, *p* < 0.001, η_p_^2^ = 0.16.

As hypothesized (H4a), participants frequently referenced extra material resources (*M* = 0.48, *SD* = 0.50) and shared identity (*M* = 0.22, *SD* = 0.42). Participants referenced extra material resources significantly more than shared identity *t*(81) = 2.91, *p* = 0.005, *d* = 0.56, and moral reasons (*M* = 0.07, *SD* = 0.26), *t*(81) = 5.82, *p* < 0.001, *d* = 1.03. Participants also referenced shared identity significantly more than moral reasons, *t*(81) = 2.53, *p* = 0.01, *d* = 0.43.

Participants then viewed a low‐wealth Indian group who could choose a same‐wealth (low‐wealth) Indian friend, or an other‐wealth (high‐wealth) Indian friend. Overall, more participants expected the group to prefer the other‐wealth (high‐wealth) friend. A one‐way repeated measures ANOVA was performed to compare the usage of the reasoning categories (moral reasoning, positive appearances or traits, recognition of extra material resources) by participants who demonstrated this expectation. The repeated measures ANOVA revealed a significant difference between the reasoning categories used, *F*(2) = 33.20, *p* < 0.001, η_p_^2^ = 0.35. Participants referenced extra material resources (*M* = 0.60, *SD* = 0.50) significantly more than positive appearances or traits (*M* = 0.06, *SD* = 0.25), *t*(61) = 6.76, *p* < 0.001, *d* = 1.37, and significantly more than moral reasons (*M* = 0.10, *SD* = 0.30), *t*(61) = 5.86, *p* < 0.001, *d* = 1.21, thus partially supporting H4b. There were no significant differences in the references to positive appearances or traits and moral reasons, *t*(61) = 0.63, *p* = 0.52, *d* = 0.14.

#### 3.2.2 Cross Condition

In the Cross Ethnicity/Wealth condition, participants either saw a high‐wealth or low‐wealth Chinese group. The high‐wealth Chinese group could choose a same‐ethnic *but other‐wealth* (low‐wealth Chinese) friend or a same‐wealth *but other‐ethnic* (high‐wealth Indian) friend. Overall, participants expected the group to prefer the same‐wealth *but other‐ethnic* (high‐wealth Indian) friend. A one‐way repeated measures ANOVA was performed to compare the usage of the reasoning categories (moral reasoning, shared identity regarding wealth, recognition of extra material resources) by participants who demonstrated this expectation. The repeated measures ANOVA revealed a significant difference between the reasoning categories used, *F*(2) = 4.35, *p* = 0.02, η_p_^2^ = 0.11. Participants referenced extra material resources (*M* = 0.39, *SD* = 0.50) significantly more than moral reasons (*M* = 0.08, *SD* = 0.27), *t*(37) = 3.14, *p* = 0.003, *d* = 0.77, supporting H4a. Participants also frequently referenced shared identity (H4a), but there were no significant differences in references to shared identity (*M* = 0.24, *SD* = 0.43) and extra material resources, *t*(37) = 1.23, *p* = 0.23, *d* = 0.32, nor moral reasons, *t*(37) = 1.78, *p* = 0.08, *d* = 0.45.

The low‐wealth Chinese group could choose a same‐ethnic *but other‐wealth* (high‐wealth Chinese) friend or a same‐wealth *but other‐ethnic* (low‐wealth Indian) friend. Overall, participants expected the group to prefer the same‐ethnic *but other‐wealth* (high‐wealth Chinese) friend. A one‐way repeated measures ANOVA was performed to compare the reasoning categories (moral reasoning, positive appearances or traits, recognition of extra material resources) by participants who demonstrated this expectation. The repeated measures ANOVA revealed no significant difference between the reasoning categories used, *F*(2) = 2.00, *p* = 0.14, η_p_^2^ = 0.05. Participants often referenced moral reasons (*M* = 0.26, *SD* = 0.44) and extra material resources (*M* = 0.23, *SD* = 0.43), supporting H4b. (See Table 7 for a summary of the three most used reasoning categories in both the Wealth and Cross conditions; see Supplemental Materials for full table.)

**TABLE 7:** Observed means and standard deviations for the top three reasoning categories used by condition and participant's choice.

Condition	Group	Friendship prediction	*N*	Moral reasoning		Shared identity		Extra material resources	
				*M*	*SD*	*M*	*SD*	*M*	*SD*
Wealth	High‐wealth Chinese	Same‐wealth	82	0.07	0.26	0.22	0.42	0.48	0.50

Condition	Group	Friendship prediction	*N*	Moral reasoning		Positive appearances/traits		Extra material resources	
				*M*	*SD*	*M*	*SD*	*M*	*SD*
Wealth	Low‐wealth Indian	Other‐wealth	62	0.10	0.30	0.06	0.25	0.60	0.49

Condition	Group	Friendship prediction	*N*	Moral reasoning		Shared identity		Extra material resources	
				*M*	*SD*	*M*	*SD*	*M*	*SD*
Cross	High‐wealth Chinese	Same‐wealth but *other‐ethnic*	38	0.08	0.27	0.24	0.43	0.39	0.50

Condition	Group	Friendship prediction	*N*	Moral reasoning		Positive appearances/traits		Extra material resources	
				*M*	*SD*	*M*	*SD*	*M*	*SD*
Cross	Low‐wealth Chinese	Same‐ethnic but *other‐wealth*	39	0.26	0.44	0.08	0.27	0.23	0.43

*Note*: By design, participants viewed only one of the Cross conditions; thus, the *n*s were 50% of the sample. In each condition, participants were excluded if they gave uncodable responses.

## 4 Discussion

This study demonstrated novel findings regarding young Chinese Singaporean children's awareness that peer groups make inclusion decisions based on social status indicators such as ethnicity and wealth. Past research has shown that young children often display a same‐race or same‐ethnicity bias (Gonzalez et al. 2017; Singh et al. 2022; Qian et al. 2015, 2019; Rizzo et al. 2022; Xiao et al. 2014) and more recently that young children also display a high‐wealth bias (Ahl et al. 2019; Horwitz et al. 2014; Shutts et al. 2016). A novel contribution of this study is that, beyond their own preferences and biases, children as young as 4–7 years old expected their peers to act on these biases when selecting a new peer to join their group. Additionally, the reasoning children used to justify their predictions of groups’ inclusion decisions reveals that children recognize the asymmetry of ingroup preferences for high‐ and low‐wealth groups beginning early in development. These findings extend prior work with older children in the US (Burkholder et al. 2021) by demonstrating that expectations about how peer groups consider ethnicity and wealth are already emerging in early childhood. However, the patterns observed in the present study differ from those documented in Burkholder et al. (2021), suggesting developmental changes or cultural differences in how children integrate multiple social status cues when reasoning about expectations for group inclusion.

Children in the present study expected both Chinese and Indian peer groups to include a new peer that matched the group's ethnicity (when wealth was held constant), and this expectation increased with age. Although young children, including Chinese Singaporean children, have been shown to display preferences for their ethnic ingroup (Lee and Setoh 2022; Kinzler and Spelke 2011; Lam et al. 2011; Setoh et al. 2019), the children in our study did not impose their own preferences onto other‐ethnic children. That is, they did not expect all groups to prefer to include a Chinese friend over an Indian friend. Instead, they expected an Indian group of friends to choose a new Indian friend over a Chinese one, as would be expected by the SRD model. This extends previous research on 6‐ to 8‐year‐old Australian children's awareness of group norms and recognition that other groups may have different preferences (Nesdale 2008), as well as past research conducted with older American participants showing that majority‐ethnic children view same‐ethnic inclusion as more likely to occur than interracial inclusion (Cooley et al. 2019). Importantly, we document these findings in the understudied context of Singapore.

Our main findings from the Wealth and Cross Ethnicity/Wealth conditions indicated that participants mostly expected peer groups to prioritize same‐wealth friendships when the group was high‐wealth. When only wealth was varied, we found that children expected both low‐wealth and high‐wealth groups to choose a high‐wealth friend. However, with age, children expected both groups to prefer a same‐wealth friend. When both ethnicity and wealth were independently manipulated, participants prioritized same‐wealth friendships when the group was high‐wealth but prioritized other‐wealth (high‐wealth) friendships when the group was low‐wealth (see Table 6). In contrast to the Wealth condition, no age‐related changes were observed in the Cross Ethnicity/Wealth condition.

One possible explanation is that children's tendencies to expect same‐wealth friendships with age were counterbalanced by their concurrent expectations for same‐ethnicity preferences. Indeed, in the Ethnicity condition, children increasingly expected groups to favor same‐ethnicity peers with age. Thus, when wealth and ethnicity were varied simultaneously, these competing influences may have offset one another, resulting in no detectable age effects. Taken together, these findings demonstrate a clear high‐wealth preference in children's predictions of the groups’ choices by shifting from choices in inter‐ethnic and same‐ethnic peer contexts.

Past research has shown that majority‐ethnic US White children ages 8–14 years expect groups to give priority to same‐wealth peers over same‐race peers (Burkholder et al. 2021). Given that participants in the Burkholder et al. (2021) study were older than those in the current study, high‐wealth (rather than same‐wealth) preferences may evolve with age. Another possibility is that these preferences are culturally mediated. Singaporean cultural norms emphasize education and material success (see Koh 2014; Luo et al. 2014). Parental attitudes could influence children's predictions about friendship inclusion choices (Grütter et al. 2021; Gunderson et al. 2012). Further, Lam et al. (2011) has demonstrated the effects of parental attitudes on children's biases in other contexts. In Singapore, a commonly used linguistic term “kiasu,” encapsulates this norm. Often applied to educational and occupational success, this term refers to a cultural norm of competitiveness and fear of missing out on coveted opportunities and material success. For example, in comparison to their American counterparts, Chinese Singaporean adolescents have been found to have higher levels of anxiety (Li et al. 2008) and greater academic stress (Ang et al. 2007), given societal and educational pressures which emphasize competitiveness and meritocracy (Zhou and Wang 2019). Thus, cultural norms could have led children in our study to choose a peer that was high‐status with more material resources, even if that could threaten the cohesiveness or identity of the group. Future research should examine parental values as a contributor to children's views about wealth status in contexts that reflect different sociopolitical messages and include a wider age range for the child sample than the one examined in the present study. The age of the child and parental messages could be analyzed to disentangle age and sociocultural messages as significant contributors to predictions about the priority assigned to wealth status in the context of friendship inclusion.

The SRD model provides a framework for understanding children's social reasoning in intergroup contexts (Elenbaas et al. 2020; Rutland et al. 2024). In this study, participants often justified choosing a high‐wealth peer over a low‐wealth peer by referencing the high‐wealth peer's access to extra material resources and opportunities. In contexts where the high‐wealth peer was to join a high‐wealth group, the shared identity between the peer and group regarding material resources was also commonly referenced. In the Cross Ethnicity/Wealth condition, participants explained their expectations for the low‐wealth Chinese group's choice of a high‐wealth Chinese peer by referencing moral reasons such as fairness, sharing, or the benefits of diversity. Notably, participants did not frequently reference moral reasons in the Wealth condition when justifying their expectations for the low‐wealth Indian group to choose a high‐wealth Indian peer. This difference in expecting the high‐wealth Chinese group to engage in moral behaviors such as sharing resources to a greater extent than the high‐wealth Indian group could partially be attributed to participants’ own‐ethnic biases and overall positive view of their group. The current findings reflect the SRD theory's assertion that children do not consistently or indiscriminately endorse in‐group inclusion but rather flexibly consider different factors when evaluating social decisions (Burkholder et al. 2021). These findings provide support for this theory as children as young as 4–7 years of age were shown to appreciate the group's identity when choosing a high‐wealth peer for a high‐wealth group, but would reference moral reasons (e.g., sharing to benefit the welfare of the group) when choosing a high‐wealth peer for a low‐wealth group (in cases when the peer was Chinese).

Children demonstrated an expectation that high‐wealth peers would share their resources with a low‐wealth group or at least benefit the group via their extra resources and opportunities. However, when the group was high‐wealth, participants did not expect the group to pick a low‐wealth peer and share their resources with them. In sum, children expect peer groups to maximize their self‐interest and they do so by prioritizing high wealth in peer inclusion decisions. The SRD framework seeks to find out how the nature of intergroup settings can impede or advance children's decisions and positive outgroup attitudes (Cooley et al. 2019). These findings extend SRD by demonstrating that when the group is established to be in an advantageous position (i.e., high‐wealth status), children's moral considerations are impeded and not consistently applied.

### 4.1 Broader Impacts and Implications

Our findings suggest that young children expect peer groups to prioritize high‐wealth status over ethnicity when making inclusion decisions. In everyday life, ethnicity and wealth are commonly confounded, and ethnic bias is not as predictable or consistently activated as previous literature suggests (e.g., Gonzalez et al. 2017; Qian et al. 2015, 2019; Xiao et al. 2014). This highlights a need for intervention strategies that aim to reduce social bias by considering multiple aspects of group membership, a goal which aligns with recent discussions in developmental science on the importance of applying intersectional lenses to children's social cognition (Lei and Rhodes 2021).

Minoritized groups tend to be associated with low‐wealth status or to have experienced discrimination in the past that resulted in being disadvantaged when it comes to wealth accumulation over several generations (Kaushal and Nepomnyaschy 2009; Parker and Stovall 2004). Wealth serves as a primary marker of status in many societies and is often intertwined with ethnic identity. Research indicates that even young children can discern these hierarchies and understand how different social markers contribute to them (Amemiya et al. 2023; Heck et al. 2022; Olson et al. 2012). As such, wealth disparities are aggregated for minoritized ethnic groups, and Singapore is no exception. Biases toward racial and ethnic minorities could develop from or be strengthened by assumptions that they are of low‐wealth status. Given the clear high‐wealth preference our participants expected peer groups to hold, this is especially important to tackle. This is supported by past research that explicit preferences for high‐status individuals predicted implicit biases favoring the majority‐ethnic group that was associated with high status and wealth in that society (Newheiser et al. 2014). At the same time, research has also shown that beginning in middle childhood through adolescence, an awareness of systemic biases and wealth inequalities emerges for some whereby a desire to rectify wealth inequalities is often expressed (see Elenbaas 2019: US; Gonul et al. 2023: Türkiye; Grütter et al. 2021: Nepal).

Research on intervention strategies to reduce social bias has focused on mitigating ethnic bias in children through positive out‐group exemplars (Gonzalez et al. 2017, Gonzalez et al. 2021), perceptual individuation training (Lebrecht et al. 2009; Qian et al. 2015, 2019), parental involvement (Scott et al. 2020) and teacher‐led discussions at school (Killen et al. 2022). Our focus on children's expectations about group norms provides a window of opportunity for change. Intervention studies in schools that promote conversations about the reasons for social inclusion and exclusion enable children to recognize that exclusive group norms are not necessarily shared by everyone. In guided classroom discussions, children have the opportunity to challenge unfair group norms, as well as hear their peers do so. As an example, in a school‐based intervention program, *Developing Inclusive Youth* (Killen et al. 2022), children ages 8–11 years engaged in a teacher‐led classroom discussion about social exclusion based on various group identities (e.g., race, ethnicity, gender, wealth status, immigrant status) after watching a short online vignette about peers discussing whether to include or exclude. Significant outcomes were documented with a post‐test survey whereby children were more likely to have positive rather than negative expectations of diverse peers. The current findings suggest that this type of program could be effective for younger children, ages 4–7. Our study emphasizes a need to also incorporate measures focused on wealth biases, which are tied to ethnic biases, when developing intervention methods.

### 4.2 Limitations and Future Directions

The present study yielded novel findings regarding children's developing understanding of group knowledge about norms involving ethnic and wealth biases. Many new avenues of research could be conducted to extend this research and shed light on some of the interpretations provided. This study consisted of a demographically underrepresented sample in the developmental science literature, with its focus on Chinese Singaporean children. Nonetheless, our participants were from middle‐ to high‐income households. Investigating how children from a wider range of socioeconomic backgrounds make predictions about peer group inclusion preferences, while considering their perception of their own wealth level or social status, would provide insight into how diverse perceptions or experiences of wealth impact how children make or evaluate decisions for groups of the same or differing wealth levels.

One limitation in our study was that a notable proportion of children did not provide a codable response when asked to explain why a group would prefer a same‐ethnicity or other‐ethnicity peer in the Ethnicity condition. Children often did not respond or said “I don't know”. This limited our ability to fully assess children's reasoning about expectations for expected ethnicity‐related in‐group biases. We recommend that future research focus specifically on children's reasoning about ethnicity, particularly regarding their expected perceptions of group‐based bias and consider strategies to elicit more complete responses. Moreover, as limited research has examined young children's expectations about peer inclusion preferences in these contexts, our reasoning analyses primarily aimed to characterize how young children reason about these issues rather than to examine age‐related changes across the 4–7‐year range. Future research should therefore investigate how reasoning about group norms and biases develops across early and middle childhood.

Additionally, all participants were Chinese, the majority‐ethnic group in Singapore. An important next step would be to include the perspectives of minority‐ethnic groups in Singapore, such as Indian Singaporean children, who do not show strong own‐ethnic biases in the way that has been displayed by Chinese Singaporean children (Setoh et al. 2019), and Malay Singaporean children, who, to our knowledge, have not been studied in this context. On one hand, children from minority‐ethnic groups in Singapore could have experienced unfair treatment or discrimination and could thus expect friendship groups to prioritize choosing a peer from the majority‐ethnic group (i.e., Chinese), as compared to choosing a same‐ethnicity or same‐wealth or high‐wealth peer.

On the other hand, children from minority‐ethnic groups in Singapore could be equally influenced by the competitive culture that values material success and could expect groups to prioritize high‐wealth peers, similar to the Chinese children in the present study. Given that the expression of ethnic bias can differ in majority and minority groups, it would be important to examine whether this would impact wealth biases and whether the prioritization of ethnicity and wealth in children's friendship choices would differ. Further, future research should contextualize demographic variables according to the specific settings where the data were collected. Markers of wealth can vary significantly between different ethnicities and countries, and these contextual differences should be taken into account.

### 4.3 Conclusion

This study found that Chinese Singaporean 4‐ to 7‐years‐olds demonstrated knowledge that peer groups make inclusion decisions based on wealth and ethnic preferences. Participants consistently expected peer groups to express high‐wealth preferences and were also flexible in using different concepts to justify their predictions depending on the group's characteristics, demonstrating the complexity of their reasoning even at this young age. Revealing children's awareness of group preferences that reflect biases at such an early age indicates that intervention programs are urgently needed. Children who experience social exclusion based solely on their ethnicity, wealth status, or both are at risk for anxiety, stress, and depression (Cheng et al. 2015; Currie et al. 2019; Rivas‐Drake et al. 2014). The present study reveals important targets for intervention based on children's perceptions of factors that determine inclusion and exclusion. Given that ethnicity and wealth are closely interconnected, our findings suggest that school curricula programs and intervention research consider investigating children's predictions, evaluations, and reasoning about wealth and ethnicity to mitigate biases and prejudices that emerge early in child development.

Attitudes about social exclusion emerge early in childhood, often contributing to prejudicial attitudes. If left unchanged, these attitudes become deeply entrenched and difficult to change by adulthood. Adult relationships in the workforce need to be congenial, cooperative, and collaborative to be productive, and for creating a civil society. Thus, reducing exclusionary attitudes early in life contributes to sustainable development across cultural contexts.

## Conflicts of Interest

We have no known conflicts of interest.

## Supporting information




**Supporting File 1**: desc70175‐sup‐0001‐SuppMat.docx

## Data Availability

The data and the code will be available upon request from the corresponding author; this study was not preregistered.

## Endnotes

1 Models with random intercepts for items as well as participants were also fit. However, including random intercepts for participants led to model convergence issues. Additionally, model comparison procedures using likelihood ratio tests indicated that including random intercepts for items did not significantly improve model fit. Thus, random intercepts for items were not included in the final models.
