# Supplementary material for: Chinese Singaporean Children's Expectations About Peer Group Norms in the Context of Wealth and Ethnicity
Source: Dev Sci. 2026 Mar 24;29(3):e70175. doi: 10.1111/desc.70175 (PMC13010226; doi:10.1111/desc.70175)
Supplement: Supplementary file 1 — Supporting File 1: desc70175‐sup‐0001‐SuppMat.docx [file DESC-29-e70175-s001.docx]

Chinese Singaporean Children’s Expectations about Peer Group Norms

in the Context of Wealth and Ethnicity

**Supplemental Materials**

**Parent and Child Reports of Participants’ Diverse Peer Relationships**

To determine children’s extent of experience with other-ethnic peers, parents, and participants were asked about their social relationships with their own- and other-ethnic peers. Participants and their parents were asked whether the participants had friends in four ethnic groups: Chinese, Malay, Indian, and Caucasian. Participants were shown pictures of children in each of the ethnic groups and asked if they had friends like them. Data from 12 participants could not be obtained due to a misunderstanding of the task. Of the remaining 91 participants, 92% reported having own-ethnic friends and 100% of their parents reported that their children had own-ethnic friends. For the other three ethnic groups, the mean reported friendships by children was 33% for Indian, 48% for Caucasians, and 57% for Malay, with an 83% match to the parent’s report. Thus, the vast majority of Chinese participants had same-ethnic Chinese friends.

**Preparation of Stimuli**

To ascertain that the photos of the individual children used in the vignettes were accurate representatives of their ethnic group and were comparable in terms of attractiveness and perceived friendliness across ethnic groups, all photographs used were rated by 10 Singaporean Chinese young adults (age range: 21–24 years). They were asked to identify the child’s ethnicity and rate attractiveness and friendliness on a Likert scale of 1 to 7. The adults correctly identified the children’s ethnicity 100% of the time. On attractiveness, the average ratings for photographs of Chinese children (*M* = 4.86, *SD* = 0.38) were not significantly different than those of Indian children (*M* = 4.94, *SD* = 0.38), *t*(34) = 0.66, *p* = .52, *d* = 0.22. Similarly, on friendliness, the average ratings for photographs of Chinese children (*M* = 5.28, *SD* = 0.42) were not significantly different than those of Indian children (*M* = 5.49, *SD* = 0.43), *t*(34) = 1.50, *p* = .14, *d* = 0.51. Furthermore, t-tests were done to ensure that within each scenario, the two friends from which the group could choose did not significantly differ in attractiveness and friendliness ratings.

**Observed Means and Standard Deviations for Reasoning Categories Used by Condition and Participant’s Choice**

| Condition | Group | Friendship prediction | *N* | Moral reasoning | | Shared ethnicity/skin color | | Shared identity regarding wealth | | Shared identity regarding language or traditions | | Positive appearances/ traits | | Extra material resources | | Uncodable | |
| --- | --- | --- | --- | --- | --- | --- | --- | --- | --- | --- | --- | --- | --- | --- | --- | --- | --- |
|  |  |  |  | *M* | *SD* | *M* | *SD* | *M* | *SD* | *M* | *SD* | *M* | *SD* | *M* | *SD* | *M* | *SD* |
| Wealth | High-wealth Chinese | Same-wealth | 82 | 0.07 | 0.26 | 0.01 | 0.11 | 0.22 | 0.42 | 0 | 0 | 0.05 | 0.22 | 0.48 | 0.5 | 0.17 | 0.38 |
| Wealth | Low-wealth Indian | Other-wealth | 62 | 0.1 | 0.3 | 0.02 | 0.13 | 0 | 0 | 0 | 0 | 0.06 | 0.25 | 0.6 | 0.49 | 0.23 | 0.42 |
| Cross | High-wealth Chinese | Same-wealth but other-ethnic | 38 | 0.08 | 0.27 | 0 | 0 | 0.24 | 0.43 | 0 | 0 | 0.08 | 0.27 | 0.39 | 0.5 | 0.21 | 0.41 |
| Cross | Low-wealth Chinese | Same-ethnic but other-wealth | 39 | 0.26 | 0.44 | 0 | 0 | 0 | 0 | 0 | 0 | 0.08 | 0.27 | 0.23 | 0.43 | 0.44 | 0.5 |

*Note.* By design, participants viewed only one of the cross conditions; thus the *n*s were 50% of the sample. In each condition, participants were excluded if they gave uncodable responses.

**Summary of Hypotheses and Central Findings**

| Hypotheses | Findings | Hypothesis confirmed? |
| --- | --- | --- |
| H1a: For both Chinese and Indian friend groups, participants will expect the group to choose the same-ethnic peer over the other-ethnic peer. | Participants expected both the Chinese and Indian friend groups to choose the same-ethnic peer over the other-ethnic peer. | Yes |
| H1b: The expectation for choosing a same-ethnic peer will increase with age. | With age, participants increasingly expected the Chinese and Indian friend groups to choose the same-ethnic peer over the other-ethnic peer. | Yes |
| H2a: For both a high- and low-wealth friend group, participants will expect the group to choose the high-wealth peer over the low-wealth peer. | Participants expected both the high- and low-wealth friend groups to choose the high-wealth peer over the low-wealth peer. | Yes |
| H2b: The expectation for choosing a same-wealth peer will increase with age. | With age, participants increasingly expected the high-wealth and low-wealth friend groups to choose the same-wealth peer over the other-wealth peer. | Yes |
| H3: When varying both wealth and ethnicity, participants will expect both a low- and high-wealth Chinese friend group to choose a high-wealth peer, regardless of ethnicity. | Participants expected both the low- and high-wealth Chinese friend groups to choose the high-wealth peer over the low-wealth peer, regardless of ethnicity. | Yes |
| H4a: Participants would reference access to extra material resources and shared identity when explaining why they thought a high-wealth group would pick a high-wealth peer. | Participants largely thought that high-wealth groups prefer to include high-wealth peers because they possess extra material resources and because of shared identity regarding wealth. | Yes |
| H4b: Participants would reference access to extra material resources and moral reasons when explaining why they thought a low-wealth group would pick a high-wealth peer. | Participants largely thought that low-wealth groups prefer to include high-wealth peers because they possess extra material resources.  Children additionally expected a low-wealth Chinese group to prefer a same-ethnic other-wealth (high-wealth Chinese) peer because a high-wealth peer can share those resources with the low-wealth group, as suggested by the use of moral reasoning. However, this reasoning was not evidenced when participants predicted a low-wealth Indian group’s choice for including a high-wealth Indian peer. | Yes, partially |
